# Mentees perception of Formal Mentorship at public sector Medical School, Pakistan

**DOI:** 10.12669/pjms.41.2.11055

**Published:** 2025-02

**Authors:** Tayyiba Wasim, Fatima Haroon, Afshan Shahid, Anaab Wasim

**Affiliations:** 1 Tayyiba Wasim, FCPS, FRCOG, Department of Obstetrics and Gynaecology, Services Hospital, Lahore, Pakistan; 2 Fatima Haroon, FCPS, Department of Obstetrics and Gynaecology, Services Hospital, Lahore, Pakistan; 3 Afshan Shahid, FCPS Department of Community Medicine, Services Institute of Medical Sciences, Lahore, Pakistan; 4 Anaab Wasim, MBBS student Lahore Medical & Dental College, Lahore, Pakistan

**Keywords:** Mentorship program, Mentee perceptions, Strengths and weaknesses

## Abstract

**Objective:**

To evaluate medical students’ experiences with mentors along with strengths and limitations of the first formal mentorship program at Medical School.

**Methods:**

This cross sectional study was conducted at Services Institute of Medical Sciences, a public sector medical college in Lahore. The mentoring program was planned and implemented for first time from January 2022 to November 2022 for MBBS students. Mentors were faculty members who received training prior to the program. Mentorship session of two hours every month was included in timetable. After the successful completion of one year of the program, the feedback questionnaire was filled by mentees about their perceptions about mentors and mentorship program and analyzed.

**Results:**

A total of 362 students gave feedback. The findings highlight that 327/362 (90.3%) of students had positive experiences with their mentors, particularly in areas of mentor accessibility, confidence building, learning ethical behavior, how to study smart, professionalism, psychological support and communication skills. There were five key strengths identified by student responses including personal development and support 141(40%), better communication and interaction 131(35%), time and relationships management skills 22(6%) and professional guidance 10(3%). Around 107(29%) of the students did not perceive any weaknesses in the mentorship program. Half of the respondents expressed concerns about the lack of assurance regarding confidentiality. Around (15.1%) reported hesitancy in communicating within group settings and suggested individual distraction free sessions.

**Conclusion:**

Formal mentorship program is beneficial in personal and professional development of students. A comprehensive framework that ensures confidentiality should be considered to enhance its effectiveness.

## INTRODUCTION

Mentoring involves a relationship where a more experienced individual (mentor) provides guidance and trusted advice to a less experienced individual (mentee). Medical school mentoring programs have been established globally, each with different goals and objectives. Formal mentorship programs have provided medical students with increased research opportunities, career guidance, and support for both professional and personal development.^1^

As medical education continues to grow, mentors remain essential to student training and professional career development. They offer valuable wisdom, share expert insights, and help mentees develop skills for long-term success.^2^ Mentoring programs have been linked to various faculty benefits, such as enhanced recruitment, increased engagement, successful faculty promotions, improved retention rates, earlier career achievements, and positive perceptions of the institution’s commitment to its faculty.^3^

Medical schools around the world have implemented both formal and informal mentoring programs for their students. Mentoring or counseling medical students is a fundamental requirement for medical school accreditation according to national and international standards. However, in some countries, only 30% to 60% of medical schools offer formal mentoring programs.^4^ There are notable challenges in creating and implementing effective mentorship initiatives especially in low middle income countries such as time constraints for faculty and students, lack of financial and professional incentives for faculty members and lack of structured curriculum.^5^,^6^

Medical education in Pakistan has seen significant challenges over the past few decades. The combination of academic pressure, long hours, and high expectations often lead to mental health issues such as depression, anxiety, and burnout in students.^7^ High student-to-teacher ratios can limit individual attention and the quality of education. Student engagement by teachers and providing them guidance is extremely important in this digital era where student teacher distance is increasing day by day. There is a lack of structured mentorship program in Pakistan medical schools that can guide students in their clinical practice and career choices.

There are 172 medical schools in Pakistan but formal mentoring programs are sparse.^8^-^10^ First formal mentorship program for medical students was started at Services institute of Medical Sciences, a public sector medical school at Lahore Pakistan. The aim of this study was to evaluate medical students’ experiences with the benefits along with strengths and limitations of the mentoring program designed to prepare them for their roles as junior doctors. This could inform the development of a potential mentoring model for other institutions.

## METHODS

**Fig.1:**
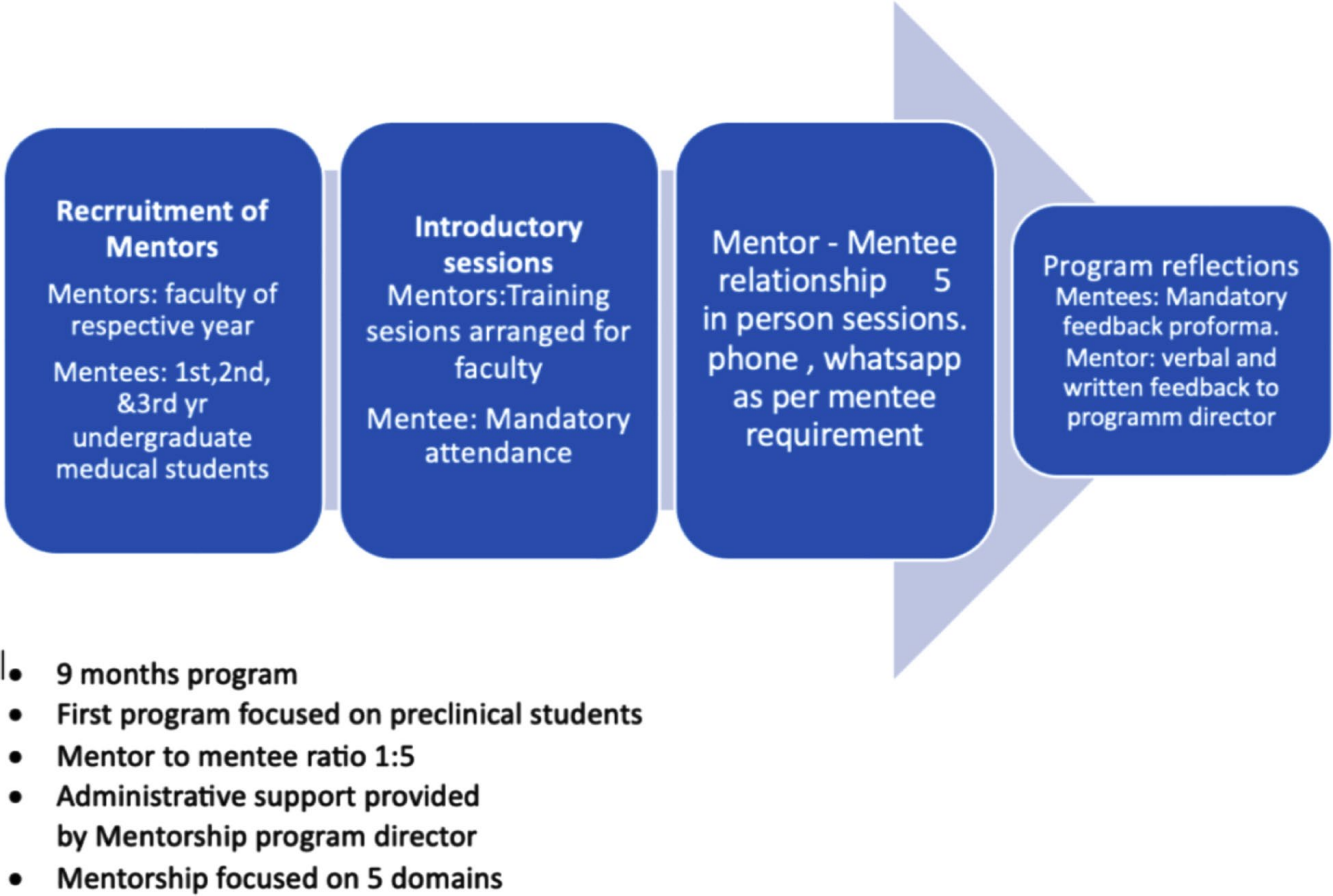
Process overview of Mentorship Program.

This cross sectional study was conducted at Services Institute of Medical Sciences which is a public sector medical college in Lahore and caters to students in five years MBBS program.The mentoring program was planned and implemented for first time from January 2022 to November 2022 to students of basic/preclinical medical sciences enrolled in 1^st^ to 3^rd^ year. Formal Mentorship program was started across all basic sciences departments. Core group of faculties including professors of all classes were notified. Mentors were faculty members from all basic science department including lecturers, assistant professors, associate professors and Professors. Mentees were assigned in 1:5 to each mentor. Training sessions of mentors were taken by experienced faculty including academic Dean and program director. Mentorship session of two hours every month was included in timetable. Mentees were guided for personal and professional development, along with ideas how to study smart, ethics, professionalism, work life balance, communication skills and future career planning. Process overview of mentorship program is shown in Fig.1.

### Ethnical Approval:

The study was approved by the institution’s Ethics Committee approval Ref no. IRB/2024/1369/SIMS dated 14.06.2024.

A structured feedback Form was developed with face and content validation carried out by the curriculum Committee in collaboration with the Medical Education department. Reliability analysis was conducted to assess the internal consistency of the questionnaire, which was measured using Cronbach’s alpha. Items were considered to have an acceptable level of internal consistency if the Cronbach’s alpha value ranged from 0.5 to 0.7, and a good level if it exceeded 0.7. The feedback form included a section of demographic profile, three open-ended and 23 close-ended questions with 16 questions about the sessions and seven questions about the program. A total sample of 362 students calculated by using open epi software. Simple random sampling was done by a making a sampling frame with the help of student’s college identification numbers.

After the successful completion of one year of the program, the feedback questionnaire was distributed to mentees to gather their perceptions of the mentorship program. Their responses were collected and analyzed. To avoid bias, the feedback questionnaires were distributed by non-teaching staff and collected in boxes placed in the department office to ensure confidentiality and anonymity. The feedback data was measured using a 5-point Likert scale (1 to 5), where Five indicated “Strongly Agree”. Open ended questions were asked at the end of the questionnaire focusing mainly on the strengths and areas of improvement in the mentoring program. Frequencies and percentages were calculated for quantitative variables and open-ended questionnaires responses were read and recurrent responses were pooled for suggestions, weaknesses, and strengths. Frequencies of responses were calculated by using SPSS software.

## RESULTS

In our study, mean age of students was 18-20 years with 214(59.1%) females and 148(40.8%) male students. The findings highlight that 327/362 (90.3%) of students had positive experiences with their mentors, particularly in areas such as clear expectations, accessibility, confidence building, ethical behavior facilitation, communication skills and work life balance. 179(49.4%) agreed and 163(45.0%) strongly agreed that their mentors were good listeners. Accessibility and availability of mentors also received high marks, with 200(55.2%) agreeing and 122(33.7%) strongly agreeing. Guidance regarding ethical behavior, how to study smart and professionalism was agreed(A)and strongly agreed (SA) by (A 204(56.4%) SA127(35.1%), 195(53.9%) SA101(27.9%) and 199(55.0) SA117(32.3%) of students respectively. Feedback on psychological support was positive, with 193(53.3%) of the participants agreed and 118(32.6%) strongly agreeing that they will be able to handle stress better. In terms of balancing personal and professional responsibilities, 56.9% agreed that the interaction with mentors was helpful. Regarding the overall organization of the mentorship program, 49.4% agreed and 25.7% strongly agreed that it was well-organized and it helped them achieve expected outcomes. (Table-I)

**Table-I:** Response of students to structured questionnaire for mentor ship program.

Statement	Strongly Disagree	Disagree	Indecisive	Agree	Strongly Agree
Mentor developed clear expectations of the mentoring relationship	8 (2.2)	7 (1.9)	20 (5.5)	233 (64.4)	94 (26.0)
Mentor was a good listener	4 (1.1)	5 (1.4)	11 (3.0)	179 (49.4)	163 (45.0)
Mentor was accessible and available	5 (1.4)	14 (3.9)	21 (5.8)	200 (55.2)	122 (33.7)
Mentor encouraged confidence building	4 (1.1)	9 (2.5)	19 (5.2)	201 (55.5)	129 (35.6)
Mentor stimulated creativity	6 (1.7)	18 (5.0)	92 (25.4)	195 (53.9)	51 (14.1)
Mentor helped in career planning	4 (1.1)	57 (15.7)	103 (28.5)	156 (43.0)	42 (11.6)
I learned how to study smart	5 (1.4)	28 (7.7)	33 (9.1)	195 (53.9)	101 (27.9)
Mentor helped in improving my communication skills	8 (2.2)	19 (5.2)	50 (13.8)	189 (52.2)	96 (26.5)
Mentor participated enthusiastically in mentoring activities	8 (2.2)	7 (1.9)	31 (8.6)	199 (55.0)	117 (32.3)
I learnt how to be good professional	8 (2.2)	7 (1.9)	31 (8.6)	199 (55.0)	117 (32.3)
Mentor facilitated improvement in ethical behavior	3 (0.8)	5 (1.4)	23 (6.4)	204 (56.4)	127 (35.1)
I learnt balancing personal and professional responsibilities	5 (1.4)	9 (2.5)	24 (6.6)	206 (56.9)	118 (32.6)
I will be able to manage stress better	5 (1.4)	2 (0.6)	31 (8.6)	193 (53.3)	131 (36.2)
Mentor guided about professional problems and helped to remove deficiencies	3 (0.8)	10 (2.8)	116 (32.0)	210 (58.0)	23 (6.4)
I anticipate an extended future relationship with my mentor	6 (1.7)	21 (5.8)	60 (16.6)	175 (48.3)	100 (27.6)
I would recommend mentor for future mentees	4 (1.1)	8 (2.2)	27 (7.5)	186 (51.4)	137 (37.8)
***Mentorship Program***					
Adequate introductory information was provided on the program in the orientation session	7 (1.9)	25 (6.9)	41 (11.3)	217 (59.9)	72 (19.9)
The mentor/mentee match was satisfactory	3 (0.8)	20 (5.5)	28 (7.7)	213 (58.8)	98 (27.1)
Mentoring program experience was satisfactory	6 (1.7)	15 (4.1)	28 (7.7)	233 (64.4)	80 (22.1)
Mentoring was professionally and personally benefitting	6 (1.7)	12 (3.3)	34 (9.4)	212 (58.6)	98 (27.1)
Mentoring program was well organized	11 (3.0)	37 (10.2)	42 (11.6)	179 (49.4)	93 (25.7)
Mentoring sessions were helpful for mentoring experience	7 (1.9)	15 (4.1)	41 (11.3)	208 (57.5)	91 (25.1)
Expected outcomes and objectives were achieved	8 (2.2)	30 (8.3)	59 (16.3)	193 (53.3)	72 (19.9)

The open-ended questions review provided an insight into the strengths, weaknesses and suggestions to improve the program in future.

### Strengths:

There were five key strengths identified by student responses were personal development and Support 141(40%), better communication and interaction 131(35%), enhanced time and relationships management skills 22(6%) and professional guidance 10(3%). Miscellaneous category also constituted 58(16%), which included communicating general issues related to campus and hostels covering various other elements that can enhance their learning experience (Table-II).

**Table-II:** Distribution of Perceived Strengths in the Mentorship Program for MBBS Students.

Category	Total Frequency	Percentage	subcategories	subcategory Frequencies
Personal Development and support	141	40%	Confidence Building, Problem Solving, Guidance and support, Motivation, Stress Reduction, Personal and Professional Development	58, 33, 22, 18,10
Communication and Interaction	131	35%	Interaction, Communication Skills, Listening Skills, Networking	57, 52, 12, 10
Miscellaneous	58	16 %	Communicating general campus issues, strategies to enhance Creativeness among students, communicating Hostel Issues, Healthy Environment	49, 4, 3, 2
Time and Relationship Management	22	6%	Time Management, Mentor- mentee Relationship and I/P relationships	11, 11
Professional Guidance	10	3%	Professional Life Awareness, Future Planning, Ethical Guidance	6, 3, 1
Total	362	100.0		

### Limitations and Suggestions for improvement:

Firstly, 107(29.9%) students perceived no weakness in the program. Non assurance of confidentiality and hesitancy to communicate in groups was perceived as most important weakness by 181(50%) and 55(15%) of students. Individual frequent sessions in distraction free environment, feedback with program director and emphasis on career guidance were suggested by students (Table-III).

**Table-III:** Distribution of Perceived Strengths, Weaknesses and Suggestions for the Mentorship Program for MBBS Students.

Strengths	Limitations	Suggestions
Personal development & support 141(40%) Better Teacher-student Interaction 131(35%) Miscellaneous 16% Time Management skills & I/P relationships 22(6%) Professional guidance 10(3%)	No weaknesses 107(29%) Non-Assurance of confidentiality 181(50%) Hesitancy to communicate in group 55(15.1%) Session was missed If Mentor is on leave 22(6%)	Need based individual session planning 20% Mentors availability to be assured 55(15.1%) Venue should be distraction free 55(15.1%) Feedback session with program director at the end of session 48(13.2%) Future carrier planning guidance 38(10.2%) Frequency of sessions should be improved 38(10.2%)

## DISCUSSION

Mentoring is an important educational strategy in medical education with various aims and objectives. The findings of our study emphasize that mentors play a critical role in the personal and professional development of medical students. The positive feedback regarding mentors’ ability to set clear expectations, listen effectively, and be accessible highlights the essential qualities of mentors that contribute to a successful mentoring relationship as shown in other studies as well.^11^,^12^ Personal development in terms of confidence building and ethical behavior were significant strength of our mentorship program identified by 141(40%) of the students.

Several studies have suggested that mentorship plays a crucial role in fostering professionalism, personal growth, and ensuring the well-being of students.^13^ Our students’ feedback and experiences highlight that mentorship provides benefits beyond what can be achieved in classroom. The roles of a mentor is distinct; while an educational or clinical supervisor focuses on planning, goal setting, and assessing performance against required training outcomes, a mentor supports personal development and offers psychological support through an ongoing relationship. Medical curriculum is overloaded, high stake exam and lack of emotional support makes the students lives stressful. In our study, it was seen that better communication with mentors and ability to express their issues helped them in overcoming their fears and stresses of hostel life. Studies have shown that students generally found it easier to seek personal and pastoral guidance from their mentors have less burnout.^14^,^15^

The most significant strength identified was the program’s role in fostering personal development and providing support. This suggests that the mentorship program successfully helps students grow on a personal level, offering guidance, encouragement, improved self-confidence and better decision-making skills. This aligns with existing literature that mentorship experiences play a crucial role in shaping their decisions regarding rotation selections, residency programs, fields of practice, and overall career direction.^16^,^17^ Through effective evaluation methods, we can assess its positive impact on students’ academic performance, career preparation, job satisfaction, research productivity and the program’s success. Our study highlighted the importance of mentors being good listeners, accessible, enthusiastic and willing to help. A study by Deng C emphasized that effective mentors possess qualities such as good interpersonal skills, initiative and the ability to provide constructive feedback.^18^

Our mentorship program had a positive feedback by majority (90.3%) of students. Setting up of a program for first time is quite challenging as there is no existing framework. Medical school faculty have heavy clinical, teaching, and research responsibilities, leaving little time for mentorship. Finding mentors who are both available and genuinely interested in engaging with students can be difficult. Not all faculty members have the skills required to be effective mentors. Matching mentors and mentees, building trust, training of mentors, adjusting sessions in time table were few challenges that we tried to address. These challenges are reflected in other studies as well.^2^,^10^,^19^ Training workshops for mentors, interest of institution head and faculty commitment were few reasons for the success of the program. Non assurance of confidentiality was identified as a limitation by half of the students and 15% felt hesitant to communicate in groups. Building trust between mentors and mentees is crucial for a successful mentorship program. However, students come from diverse backgrounds and may hesitate to share personal challenges or career concerns in groups. Establishing clear confidentiality guidelines, ensuring that both mentors and mentees understand them, and creating a safe space for open communication are vital to fostering trust.^20^

We plan to have more structured training for mentors on developing trust with mentees to ensure a more uniform and effective mentoring experience. The suggestions for improving the mentorship program at our institute regarding individual, frequent sessions, venue, exclusive mentor engagement and implementing regular feedback mechanisms will help us in designing future sessions. Enhancing the structure and content of mentoring workshops to better align with students’ needs has been highlighted in other studies as well.^6^,^21^,^22^ By fostering better teacher-student interactions, aiding in personal growth, improving time management, interpersonal skills and offering professional guidance, the program contributes significantly to the overall career success and well-being of the students. These strengths highlight the value of maintaining and further developing such mentorship initiatives within medical education.

## CONCLUSION

The formal mentorship program plays a vital role in supporting medical students’ personal and professional development. Enhancing confidentiality protocols, addressing communication barriers in group settings, ensuring session continuity and regular feedback are critical steps that could significantly strengthen the program.

### Authors’ Contribution:

**TW:** Conceived, designed, final manuscript editing.

**FH:** Literature search**,:** Did initial manuscript writing.

**AS:** data collection, statistical analysis

**AW** data collection, literature search.

All authors have read the final version and are accountable for the integrity of the study.
